# Association of DNA Repair Genes *XRCC1* and *APE-1* with the Risk of Cervical Cancer in North Indian population

**DOI:** 10.31557/APJCP.2020.21.7.2061

**Published:** 2020-07

**Authors:** Mark Rector Charles, Syed Tasleem Raza, Rolee Sharma, Pushpendra Pratap, Ale Eba, Manvendra Singh

**Affiliations:** 1 *Department of Biotechnology, Era’s Lucknow Medical College and Hospital, Lucknow, India. *; 2 *Department of Bioscience, Integral University Lucknow, Lucknow Uttar Pradesh, India. *; 3 *Centre of Bio-Medical Research (CMBRL), Sanjay Gandhi Postgraduate Institute of Medical Sciences, Lucknow, India. *

**Keywords:** XRCC1, APE-1, base excision repair, gene polymorphism, cervical cancer

## Abstract

**Backgrounds:**

Cervical cancer (CC) is one of the leading cause of death in women worldwide, HPV infection is the major risk factor in the disease development, 0and however other risk factor such as chemical carcinogens, genetic susceptibility and altered immune system are also a cause of the disease progression. In the light of the above statement we studied the base excision repair pathway (BER).

**Methods:**

We identified and studied the association of Single Nucleotide polymorphisms in the DNA repair genes of *XRCC1* (*Arg194Trp, Arg399G*,) and *APE-1Asp/148Glu* to the susceptibility of cervical cancer (CC) in North Indian population. In our study of cases (n=102). Controls (n=109) were recruited from among women without cervical abnormalities. Genotypes were determined by PCR-CTPP method, Taking DNA from peripheral blood in a case control study.

**Results:**

A positive association was observed between the polymorphisms of *XRCC1* genes, that is, in codons 194 (P=0.03, odds ratio (OR) =2.39, 95% confidence interval (CI)=5.2–1.1), 280 (P=0.01, OR=4.1, 95% CI=11.5–1.3) and 399 (P=0.01, OR=3.4, 95% CI=8.6–1.3) while *APE-1* genotype GG (p=0.03,odds ratio(OR)=0.2,95% confidence interval (CI)=0.97-0.004) we observed a statistically significant protective role in developing cervical cancer.

**Conclusion:**

Our results suggested that, *XRCC1* gene is an important candidate gene for susceptibility to cervical cancer. Although the sample size was small, the present study indicate a statistical association between cervical cancer and *XRCC1 SNPs*. Future studies are needed that may provide a better understanding of the association between gene polymorphism and cervical carcinoma risk.

## Introduction

Carcinoma of the uterine cervix is the major cause of cancer deaths among women worldwide (Lindsey et al., 2017); According to statistics by 2018, there were an estimated 569,847 new cases and approximately 311,365 deaths from cervical cancer (Bray et al., 2018). India alone accounts for roughly 120,000 case annually with 15.2% of the total cervical cancer deaths worldwide. It is wildly established that Human papillomavirus (HPV) is officially designated as a prime etiologic factor for cervical cancer. However, HPV infection is quite common, and, in most women, it clears up naturally in 8–12 months, it is estimated that only 1% of these women will develop cervical carcinoma. This shows that HPV infection is not the only determinant to develop cervical cancer, combination of other factors that is poorly understood is also responsible (Yang et al., 2020). Much effort has been given to HPV infection and external factors, but the role of host susceptibility to cervical carcinogenesis remains largely unknown (Pérez et al., 2013). In the light of these studies we investigated several studies and have identified that development of cervical cancer depends, to a significant extent, on inherited genetic factors (Brown and Leo, 2019). Among different genetic factors, variants of DNA repair genes are frequently correlated with a higher risk of several human solid tumours. 

Single nucleotide polymorphisms (SNPs) are correlated with various cancers, such as lung cancer, breast cancer, gastric cancer, and cervical cancer (D’Andrea, 2014). Thus, to develop an effective personalized treatment, the detection of SNPs is especially important for the susceptibility of cervical cancer. We have chosen Apurinic/apyrimidinic (AP) endonuclease and X-ray repair cross-complementing 1 (*XRCC1*) which are two key DNA repair genes involved in the base excision repair (BER) pathway (Naguib et al., 2020; Abbas et al., 2019). The APE-148 the protein encoded by the *APE1 *gene (present on chromosome 14q11.2-q12) creates a nick in the phosphodiester backbone of the AP site (at the 5’-) and leaves a 3’-hydroxyl group and a 5’-deoxyribose phosphate group flanking the nucleotide gap for the initiation of the BER (Bardia et al., 2012) *XRCC1* which acts as a central scaffolding protein and interacts directly with enzymatic factors such as ligase III, DNA polymerase β, and poly (ADP-ribose) polymerase to expedite efficient single strands break and evoke BER by binding to the damaged DNA (Konathala et al., 2016).

Common variants of *APE-1* (*Asp/148 Glu*) and *XRCC1* (*Arg194Trp, Arg280His, Arg399Gln*) polymorphism, respectively, have been identified as potential cancer susceptibility loci (Fan et al., 2013; Tell et al., 2005). The potential role of these polymorphisms in cancer development lies in the fact that these polymorphic gene variations alter the level of DNA repair proteins and also act as biomarker for chemotherapeutic response.(Chen et al., 2016; Peng et al., 2014) The *XRCC1* and *APE-1 *DNA repair genes was selected for this case control study because of their important role in maintaining genomic integrity. The aim of this association study is to identify disease susceptible gene variants with Cervical cancer in the women of Uttar Pradesh (North India). 

## Materials and Methods

This study was a hospital based case-control study, in which histological confirmed primary cervical cancer cases were recruited from the city of Lucknow (Era’s Lucknow Medical college & Hospital) while Controls were randomly selected from healthy postmenopausal women who requested gynaecological examinations. The criteria for selection included no positive findings during examination, no history of cancer. Sexual and reproductive history was obtained using a standardized questionnaire. And each participant signed an informed consent. A total of 102 histologically confirmed cervical cancer patients and 109 healthy control women were interviewed, completed the questionnaires, and consented to provide blood samples for genotyping. Blood samples from all study subjects were collected in EDTA-containing tubes. Genomic DNA was extracted from peripheral whole blood with the Qiagen extraction kit (Hilden, Germany), according to the manufacturer’s protocol. PCR amplifications for these two polymorphisms were performed in a programmable thermal cycler Bio rad.

Genotyping was based upon a duplex polymerase chain reaction technique with confronting-two-pair primer (PCR-CTPP) method. The amplified DNAs are allele-specific in their sizes, so that the DNA products can be applied directly for electrophoresis without the digestion by a restriction enzyme (Elizabeth et al., 2012). All primers were added into the same tube shown in (Table 2). PCR amplification was carried out to a total volume of 25 μl, containing approximately 100 ng of genomic DNA, 0.5 μl of each primer, 12.5 μl of Dream Taq green PCR master mix (2X) (Thermo Scientific, USA) (it contains dream Taq DNA polymerase supplied in 2X Dream Taq green buffer, dATP, dCTP, dGTP, dTTP, 0.4 mM each and 4 mM MgCl2) and 0.5 ul of H2O. Reaction conditions included initial denaturation at 94ºC for 5 min, followed by 30 cycles at 94ºC for 1 min, at 66ºC for 1 min, at 72ºC for 45s and a final extension at 72ºC for 5 min. PCR products were analysed by 2% agarose gel electrophoresis by using 50 bp Ladder. 

The sample size for both case and controls was calculated using QUANTO software, *χ*^2^ analysis were used to assess deviation from Hardy–Weinberg equilibrium and to compare the genotype/allele frequency between the patients and the controls. Odds ratios (ORs) were obtained by unconditional logistic regression analysis. All statistical analyses were carried out using the SPSS software, version 17.0 (SPSS Inc., Chicago, Illinois, USA). The OR was calculated using unconditional logistic regression for risk genotypes with the wild-type genotype as a reference. 

## Results

This study first analysed the association between polymorphism of DNA repair gene *XRCC1*. The characteristics of the study population is shown in (Table 1). The mean age of case and control are 49 and 45, respectively. Of the 102 case 93% of the cases had squamous cell carcinoma (SCC) and 7% had adenocarcinomas, while the stages of cases were n=27 (26.5%) Stage I, n= 37 (36.27%) stage II, n=24(23.52%) Stage III, n=14(13.72%) Stage IV ( Jalilvand and Karimi, 2020; Hans et al., 2013). HPV infection in cases were 91(89.73%) whereas HPV negative cases were 9 (11.26%). In the control group HPV positive cases were 17 (15.6%) negative cases 92 (84.04%). We also analysed the number of tobacco consumption in our study, we found that 18.36% were tobacco consumer and 81% non-tobacco consumer in the cases, while in control 4% were tobacco consumer and 93% non-tobacco consumer in the control. The genotype distribution and the alleles frequencies of all the *SNPs* ie *XRCC1* codon 194, *XRCC1* codon 280, *XRCC1* codon 399 and *APE* codon 148 are shown in (Table 3). Codon 194 (P=0.03, odds ratio (OR) =2.39, 95% confidence interval (CI)=5.2–1.1), 280 (P=0.01, OR=4.1, 95% CI=11.5–1.3), and 399 (P=0.01, OR=3.4, 95% CI=8.6–1.3.While APE 148 showed OR=0.2 P=0.03,95% CI= (0.97-0.04). Our result indicate genotype *XRCC1 194 *homozygous TT and AA genotype codon 280 of were statistically significant associated with the risk of cervical cancer with 2.39 and 4.18 fold, higher risk of cervical cancer respectively, APE-148 GG genotype showed reduced risk for developing cancer and have shown as protective role. The variant of *XRCC1* codon 399 was also associated with 3.44 fold higher risk in cervical cancer.

## Discussion

Several studies had been carried on DNA repair genes, which is recognized as a major determinants of cancer risk. While the association between different SNPs in these genes and various types of cancer are well studied, their precise association remains still inconclusive. With studies pointing to both increased and decreased risks (Gius et al., 2007). In the light of these conflicting data we examined the genotypic frequency of *XRCC1* gene and *APE* gene in a case-control study, The SNPs chosen for analysis were *XRCC1* (*Arg194Trp, Arg280His, Arg399Gln*) and *APE-1* Asp/148 Glu, The results indicated that the association with respect to His 280 genotype were significantly increased in relation to the relative risk of cervical cancer. A number of studies have been conducted on *XRCC1* and *APE* polymorphism to validate their role on gastric, breast cancer and other types of cancers. (Dai et al., 2015; Balkan et al., 2020) But fewer published information is available regarding the association between polymorphism of *XRCC1*, and *APE148 *gene and susceptibility to cervical carcinoma (Bajpai et al., 2016). Also, few studies from India have reported the genetic polymorphisms in the DNA repair genes. A numbers of studies have pointed out positive association of *Arg194Trp *variant of *XRCC1* (Jie Mei et al., 2014) with cancer development risk while other demonstrated a strong association of the *Arg399Gln* polymorphism, but other failed to find the significant result (Bajpai et al., 2016; Gius et al., 2007). We did not found any evidence for a combined effect of the *194Trp *and *399Gln* alleles of* XRCC1* and *CC* development but, *280His* and *399Gln* genotype of *XRCC1 *may be collectively related to cervical cancer risk. A similar observation was reported by (Bajpai et al., 2016) in cervical cancer risk in combination with *399Gln* (Mei et al., 2016). Our result indicates that the homozygous AA genotype of codon 280 and TT genotype of codon 194 are significantly associated with risk of CC. The heterozygote of only 399 codons present a significant risk (Borszéková et al., 2020).

Infection with the oncogenic variant of HPV has been established as the main cause of cancer lesion (Singh et al., 2004). An association between tobacco chewing and cervical cancer has been reported and we also found a 18% case of tobacco consumer in case as well as non-tobacco 81.% cases (Kaur et al., 2020) Our study showed that the *XRCC1* codon 194 (C>T) polymorphism was associated with both cervical precancer and cancer While no association was observed between *XRCC1* 399 (G>A) polymorphism, Whereas polymorphism in *XRCC1 194* and 280 with genotype *TT AA* allele plays a significant role as a risk modifier for *CC*. Our study also showed that an over representation of the codon 399 Gln allele in cases compared to controls, which suggested that this polymorphism may modify the risk for cancer. In our study, we found that a 3.44-fold increased risk of cancer with the codon 399 Gln polymorphism. However no statistically significant association was observed between APE -148 TG genotype. GG genotype showed a statistically significant association of protective effect for the cervical cancer with OR=0.2 and p <0.03. To date relatively few studies have carried out to examined the association of XRCC 1 194 exon, 280 and exon 399 together with the risk of cervical cancer. Our observation is in agreement with other study. Additional work is required to determine whether polymorphism do indeed lead to reduced DNA repair capacity and to identify various environmental factors that effects on the genes . The identification of polymorphism in many genes and the determination of their functional importance in cervical cancer will enable the design in model for de novo and therapy related diseases.

In summary our finding could offer evidence of the association between polymorphism in *XRCC1*gene and increases risk for cervical cancer. Our result was also in agreement with the hypothesis that genetic variation in DNA repair may contributes to the development in inherited genetic susceptibility to cervical carcinoma in studied population 

There was also some limitation in our analysis the sample size was not enough for a more concrete conclusion or to perform any subgroup analysis with respect to BER genotype and clinical stage. 

**Table 1:** General Information Pertaining of Controls and Cervical Cancer Patients Included in the Study

Variables	Control n=109	%	Case n=102	%	*P*-value
Age (years) (mean +SD)	45.37+10.4		49.1+13.1		0.68
Tobacco consumption					
Tobacco	4	4.34	18	18.35	0.001
Non -Tobacco	103	95.6	81	81.14	
HPV Infection					
HPV-Positive	17	15.6	91	89	0.001
HPV-Negative	92	84	8	11.26	

**Table 2:** Primer Sequence and Length of PCR Products

Primers	Sequence	PCR Product Size
*XRCC1-194 *	F1,5’ CCCTTTGGCTTGAGTTTTG3’ F2,5’GGCTCTCTTCTTCAGCT3' R1,5’GGGATGTCTTGTTGATCCG3’ R2,5’TGCTGGGTCGCTGGCTGTG3’	238,138,75
*XRCC1-280*	F1, 5-CCT ACG GCA TAG GTG AGA CC-3 R1, 5-TCC TGA TCA TGC TCC TCC -3 F2, 5-TCT GTT TCA TTT CTA TAG GCG AT-3 R2, 5-GTC AAT TTC TTC ATG TGC CA-3	460,205,63
*XRCC1-399 *	F1,5’- TCC CTG CGC CGC TGC AGT TTC T-3’, R1, 5’-TGG CGT GTG AGG CCT TAC CTC C-3’ F2, 5’-TCG GCG GCT GCC CTC CCA-3’ R2, 5’- AGC CCT CTG TGA CCT CCC AGG C-3’	63,04,47,222
*APE-148*	F1, 5-CCT ACG GCA TAG GTG AGA CC-3 R1, 5-TCC TGA TCA TGC TCC TCC -3 F2, 5-TCT GTT TCA TTT CTA TAG GCG AT-3 R2, 5-GTC AAT TTC TTC ATG TGC CA-3	360, 236,167

**Table 3:** Distribution of Genotypes and Allele Frequencies of *XRCC1*( *Arg194Trp, Arg280His, Arg399Gln*) and APE-148 Polymorphism in Cervical Cancer Patients and Controls

		Control (109) no(%)	Case (102) no(%)	OR	*P*- value	95% CI
*XRCC1-194*						
Genotype	CC	32 (29.53%)	21 (20.58%)	Refrence		
	CT	13 (11.92%)	11 (11.76%)	1.29	0.61	(3.41-0.49)
	TT	21 (19.26%)	33 (32.35%)	2.39	0.03	(5.20-1.10)
	CT+TT	34 (31.19%)	44 (43.13%)	1.97	0.05	(4.01-0.97)
Alleles	C	77 (70.64%)	53 (51.96%)	Reference		
	T	55 (50.45%)	77 (75.49%)	2.03	0.004	(3.33-1.24)
*XRCC1-280*						
Genotype	GG	29 ( 26.6%)	13 (12.73%)	Reference		
	GA	9 (8.25%)	7 (6.87%)	1.74	0.35	(5.67-0.53)
	AA	8 (7.33%)	15 (14.7%)	4.18	0.01	(11.58-1.32)
	GA+AA	17 (15.29%)	22 (21.56%)	2.89	0.02	(7.17-1.16)
Alleles	G	67 (61.46)	33 (32.35%)	Reference		
	A	25 (22.93%)	37 (36.25%)	3	0.001	(5.79-1.56)
*XRCC1 399*						
Genotype	GG	27 (24.77%)	14 (13.72%)	Reference		
	GA	25 (22.93%)	17 (16.66%)	1.31	0.55	(3.20-0.54)
	AA	14 (12.84%)	25 (24.5%)	3.44	0.01	(8.64-1.37)
	GA+AA	39 (35.77%)	42 (41.17%)	2.13	0.05	(4.65-0.98)
Alleles	G	79 (72.47%)	45 (44.11%)	Reference		
	A	53 (48.62%)	67 (65.68%)	2.22	0.002	(3.71-1.33)
APE 148						
Genotype	TT	42 (38.52%)	47 (46%)	Rreference		
	TG	39 (36.7%)	27 (26.51%)	0.63	0.14	( 1.18-0.33)
	GG	11 (10.1%)	3 (3.4%)	0.2	0.03	(0.97-0.04)
	TG+GG	49 (53.4%)	30 (32%)	0.55	0.05	(1.01-0.30)
Alleles	T	124 (57.8%)	122 (60%)	Reference		
	G	61 (28.1%)	58 (19.11%)	0.91	0.87	(1.50-0.62)

CI, confidence interval; OR, odds ratio, p >0.05

## References

[B1] Abbas M, Srivastava K, Imran M (2019). Genetic polymorphisms in DNA repair genes and their association with cervical cancer. Br J Biomed Sci.

[B2] Bajpai D, Banerjee A, Pathak S (2016). Single nucleotide polymorphisms in the DNA repair genes in HPV-positive cervical cancer. Eur J Cancer Prev.

[B3] Balkan E, Bilici M, Gundogdu B (2020). ERCC2 Lys751Gln rs13181 and XRCC2 Arg188His rs3218536 Gene Polymorphisms Contribute to Susceptibility of Colon, Gastric, HCC, Lung And Prostate Cancer. J BUON.

[B4] Bardia A, Tiwari SK, Gunisetty S (2012). Functional polymorphisms in XRCC-1 and APE-1 contribute to increased apoptosis and risk of ulcerative colitis. Inflamm Res.

[B5] Borszéková L, Ward TA, Chovanec (2020). XPA: DNA repair protein of significant clinical importance. Int J Mol Sci.

[B6] Bray F, Ferlay J, Soerjomataram I (2018). Global cancer statistics 2018: GLOBOCAN estimates of incidence and mortality worldwide for 36 cancers in 185 countries. CA Cancer J Clin.

[B7] Brown MA, Leo PJ (2019). Genetic susceptibility to cervical neoplasia. Papillomavirus Res.

[B8] Chen L, Liu MM, Liu H (2016). ERCC1 and XRCC1 but not XPA single nucleotide polymorphisms correlate with response to chemotherapy in endometrial carcinoma. Onco Targets Ther.

[B9] Dai Z, Shao Y, Kang H (2015). Relationship between apurinic endonuclease 1 Asp148Glu polymorphism and gastrointestinal cancer risk: An updated meta-analysis. World J Gastroenterol.

[B11] Elizabeth H, Andrew F, Kari EN (2012). The case-only independence assumption: associations between genetic polymorphisms and smoking among controls in two population-based studies. Int J Mol Epidemiol Genet.

[B12] Fan XM, Li KX, Niu SH, Fang ZH, Liu H (2013). Relationship of XRCC1 polymorphism with the risks and clinicopathological factors of cervical cancer. Zhonghua Yi Xue Za Zhi.

[B13] Gius D, Funk MC, Chuang EY (2007). Profiling micro dissected epithelium and stroma to model genomic signatures for cervical carcinogenesis accommodating for covariates. Cancer Res.

[B14] Gius D, Funk MC, Chuang EY (2007). Profiling micro dissected epithelium and stroma to model genomic signatures for cervical carcinogenesis accommodating for covariates. Cancer Res.

[B15] Hans E K, Magnar B (2013). Base excision repair. Cold Spring Harb Perspect Biol.

[B16] Jalilvand A, Karimi N (2020). Impact of polymorphism in DNA repair genes OGG1 and XRCC1 on seminal parameters and human male infertility. Andrologia.

[B17] Kaur J, Sambyal V, Guleria K (2020). Association of XRCC1, XRCC2 and XRCC3 gene polymorphism with esophageal cancer risk. Clin Exp Gastroenterol.

[B18] Konathala G, Mandarapu R, Godi S (2016). Data on polymorphism of XRCC1 and cervical cancer risk from South India. Data Brief.

[B19] Lindsey A, Torre FI, Rebecca L (2017). Global cancer in women: burden and trends. Cancer Epidemiol Biomarkers Prev.

[B20] Liu GC, Zhou YF, Su XC, Zhang J (2019). Interaction between TP53 and XRCC1 increases susceptibility to cervical cancer development: a case control study. BMC Cancer.

[B21] Mei J, Duan HX, Wang LL (2014). XRCC1 polymorphisms and cervical cancer risk: an updated meta-analysis. Tumour Biol.

[B22] Naguib M, Helwa MM, Soliman MM (2020). XRCC1 gene polymorphism increases the risk of hepatocellular carcinoma in Egyptian population. Asian Pac J Cancer Prev.

[B23] Peng Y, Li Z, Zhang S (2014). Association of DNA base excision repair genes (OGG1, APE1 and XRCC1) polymorphisms with outcome to platinum-based chemotherapy in advanced no small-cell lung cancer patients. Int J Cancer.

[B24] Pérez LO, Crivaro A, Barbisan G (2013). XRCC2 R188H (rs3218536), XRCC3 T241M (rs861539) and R243H (rs77381814) single nucleotide polymorphisms in cervical cancer risk. Pathol Oncol Res.

[B25] Singh A, Datta P, Jain SK (2004). Human papilloma virus genotyping, variants and viral load in tumors, squamous Intraepithelial lesions, and controls in a north Indian population subset. Int J Gynecol Cancer.

[B26] Tell G, Damante G, Caldwell D, Kelley MR (2005). The intracellular localization of APE1/Ref-1: More than a passive phenomenon?. Antioxid Redox Signal.

[B27] Yang S, Wu Y, Wang S (2020). HPV-related methylation-based reclassification and risk stratification of cervical cancer. Mol Oncol.

